# Supplementary material for: Controlled open-cell two-dimensional liquid foam generation for micro- and nanoscale patterning of materials
Source: Nat Commun. 2019 Jul 19;10:3209. doi: 10.1038/s41467-019-11281-y (PMC6642206; doi:10.1038/s41467-019-11281-y)
Supplement: Supplementary file 1 — Supplementary Information [file 41467_2019_11281_MOESM1_ESM.pdf]

Supplementary Information

Controlled open-cell two-dimensional liquid foam generation for micro- and nanoscale patterning of materials

Juyeol Bae, Kyunghun Lee, Sangjin Seo, Jun Gyu Park, Qitao Zhou, Taesung Kim*

Department of Mechanical Engineering, Ulsan National Institute of Science and Technology (UNIST), 50 UNIST-gil, Ulsan 44919, Republic of Korea

Correspondence:

T. Kim

Department of Mechanical Engineering

Ulsan National Institute of Science and Technology (UNIST)

50 UNIST-gil, Ulsan 44919, Republic of Korea

E-mail: tskim@unist.ac.kr

Tel.: +82-52-217-2313

Fax: +82-52-217-2409

Supplementary Figures

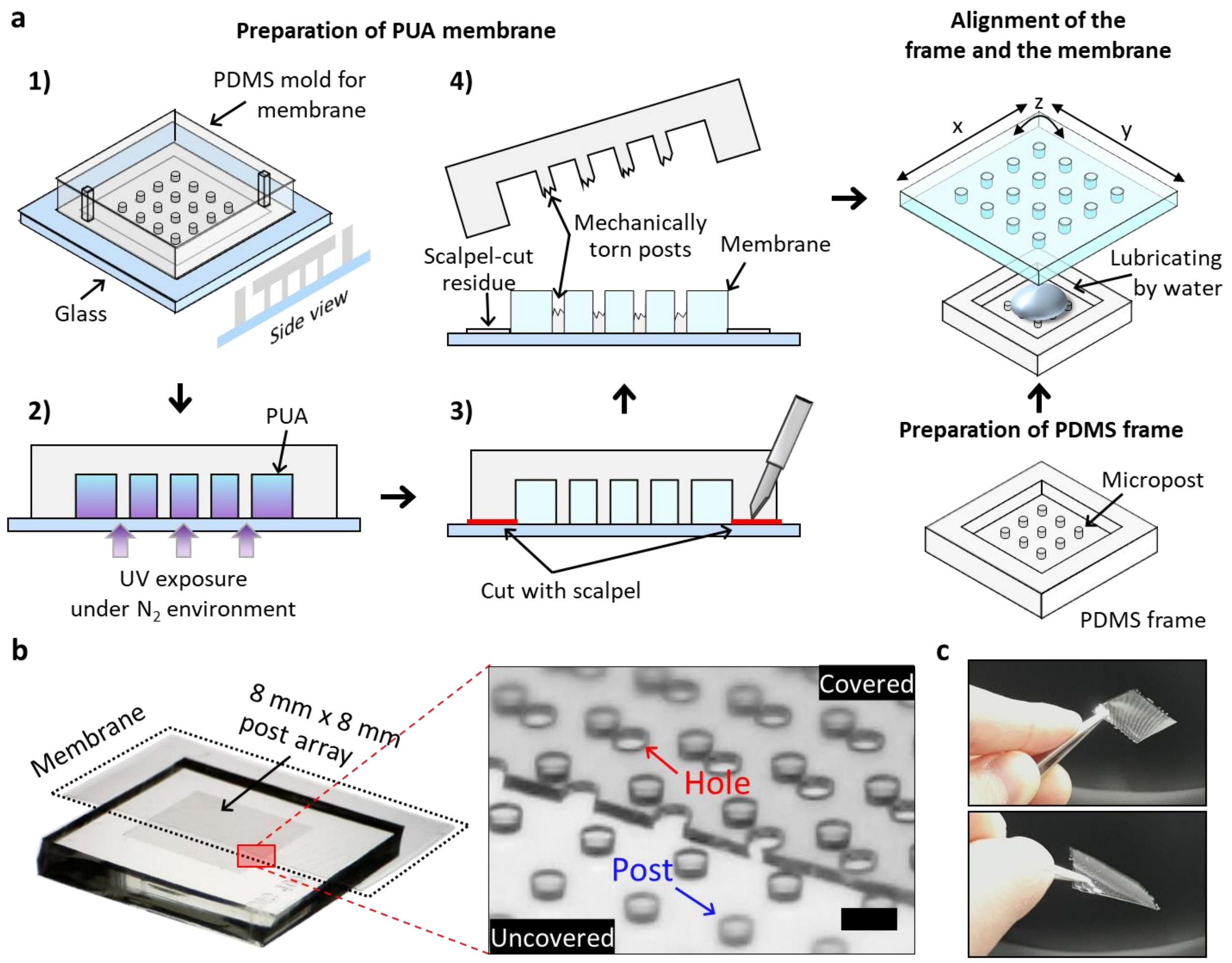

Supplementary Fig. 1. Fabrication process of the device for the MNLP. (**a**) First, PUA through-hole membrane is prepared: 1) UV-curable PUA solution is injected into the microfluidic chip fabricated using a common soft-lithography technique, 2) the PUA solution is cured under UV exposure in a nitrogen environment, 3) regions of plasma-activated bonding (bold red lines) are manually cut using a scalpel except for the post array, 4) remaining regions of plasma-activated bonding are manually torn by pulling the PDMS mold, resulting in a ruptured structure of the post array. Second, the PDMS frame is prepared using common soft-lithography. Last, for the alignment of the PDMS frame and the PUA membrane, the water layer between them is used as a lubricating layer for easy sliding during alignment. (**b**) Image of the fabricated microfluidic platform. The inset shows an enlarged view of the intentionally half-cut membrane on the PDMS frame to display the geometrical information. The scale bar is 100 µm. (**c**) Flipping the fabricated membrane upside down. The membrane was stiff enough to maintain its flatness without folding due to its high Young's modulus.

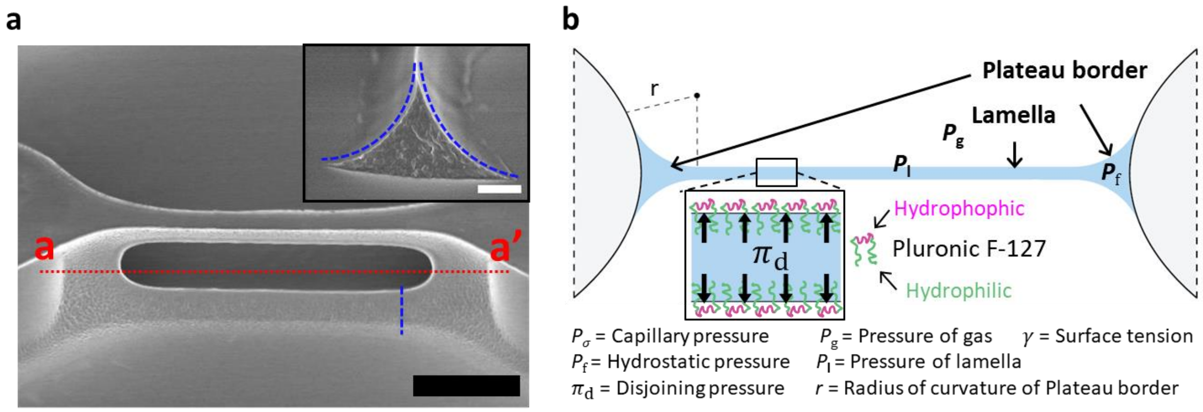

Supplementary Fig. 2. Structure of a liquid film between two neighboring microposts. (**a**) SEM image of a solidified liquid film. The top and bottom structures appear to be symmetrical when the top membrane is peeled off. The inset image shows cross section along a blue-dot line. Scale bars are 25 and 5 µm. (**b**) Illustration of the cross section along a–a’ in the top view of the SEM image. The plateau borders are adjacent to the microposts, showing curved liquid–air interfaces. A lamella is formed between the two microposts when the liquid–air interfaces get cross each other during evaporation. The thinning of lamella structure is stabilized using a surfactant. The surfactant forms a monolayer at both the liquid–air interfaces of the lamella and helps induce an intermolecular repulsive force between the two interfaces at the nanometer scale (disjoining pressure). The disjoining pressure balances with the Laplace pressure of the plateau borders and prevents the rupturing of the lamella. In other words, $\pi_d = P_l - P_f$. As $P_l \cong P_g$, $\pi_d \cong P_g - P_f = \frac{\gamma}{r}$ = Laplace pressure of the plateau border.

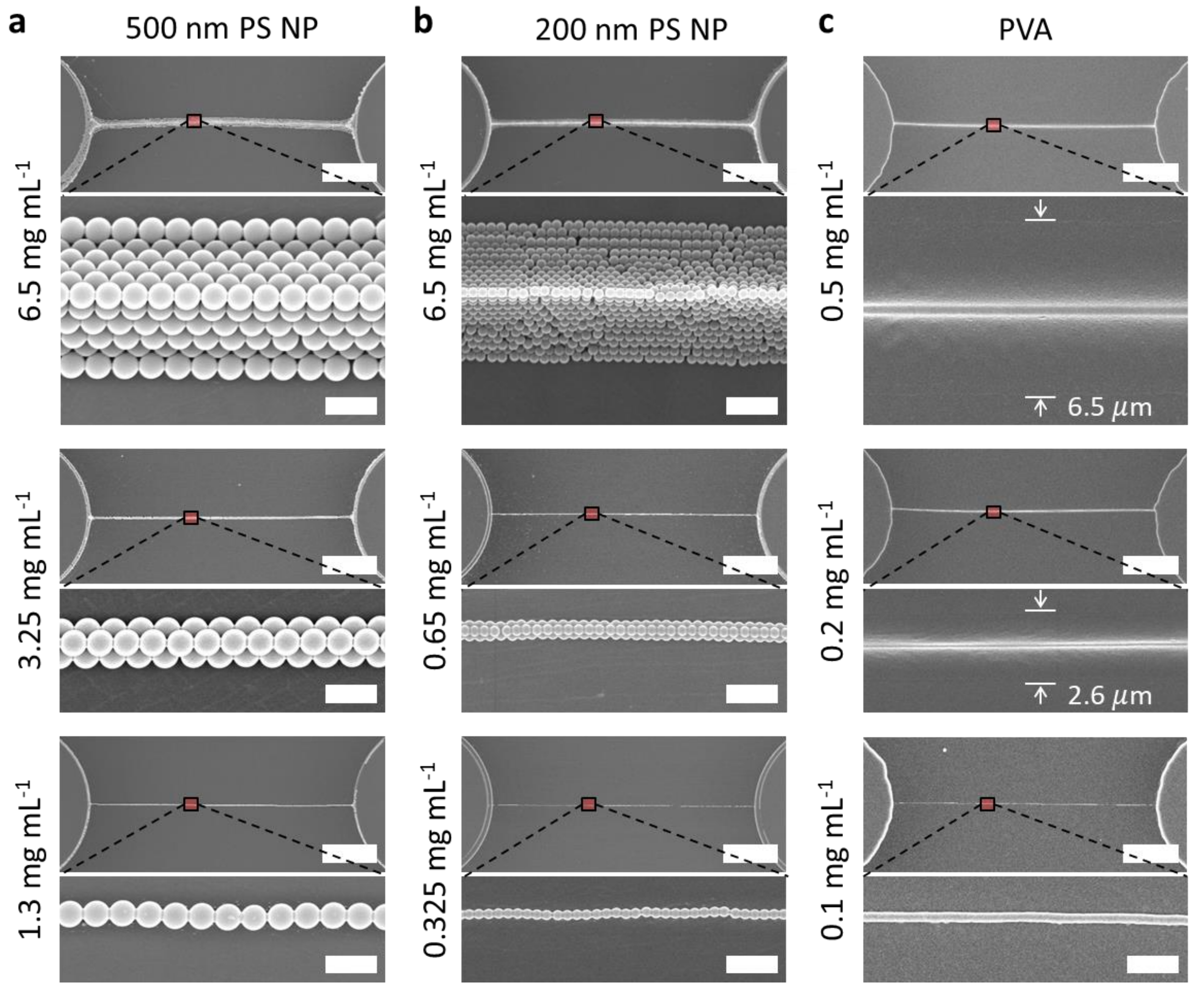

Supplementary Fig. 3. Structures of the self-assembled materials according to the concentrations of liquid samples in the MNLP. (a) Images show patterns made of 500-nm PS NP suspension at three different concentrations. After evaporation, the 500-nm PS NPs in the patterned liquid are self-assembled, forming a well-ordered particle pattern. **(b)** The same patterning process is repeated with 200-nm PS NP suspension at three different concentrations, showing the same structures as in (a). (**c**) Patterning of a solution containing PVA, forming a ridge-shaped structure between the two posts. The geometrical sizes depend on the solution concentration. The scale bars are 20 and 1 μm for the top and bottom images, respectively. Otherwise, the line width is indicated using white arrows.

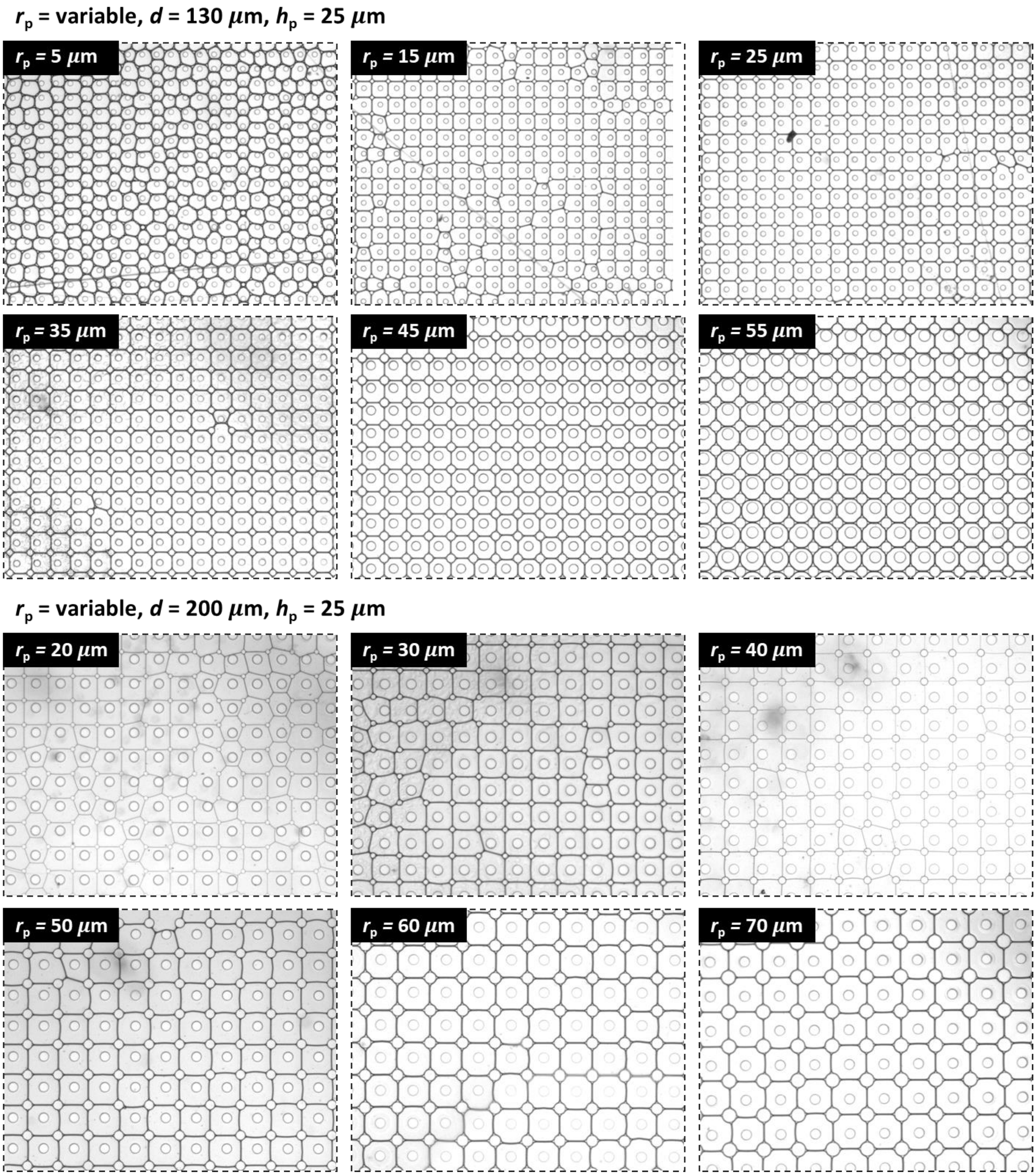

Supplementary Fig. 4. Suppression of defect generation by varying r_p values at d = 130 or 200 µm and h_p = 25 µm. At d = 130 µm and h_p = 25 µm, the optical images show results of foam generation when r_p = 5, 15, 25, 35, 45, and 55 µm, respectively. At d = 200 µm and h_p = 25 µm, the optical images show results of foam generation when r_p = 20, 30, 40, 50, 60, and 70 µm, respectively.

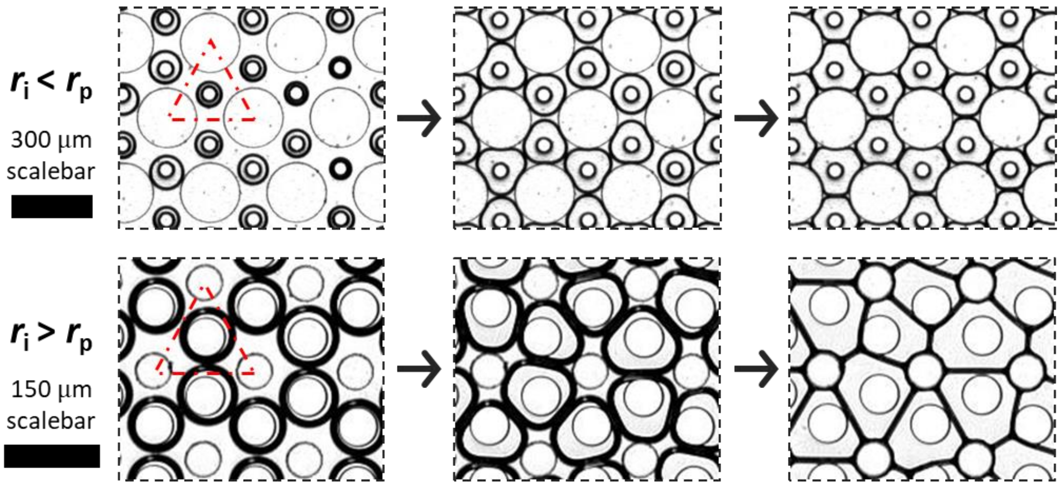

Supplementary Fig. 5. Time-sequential images of defect-free and defective pattern generations. Defect-free (top) and defective (bottom) patterns were generated with respect to the radius of the microposts in the 3P1H configuration. This is comparable with the results shown in Fig. 2b.

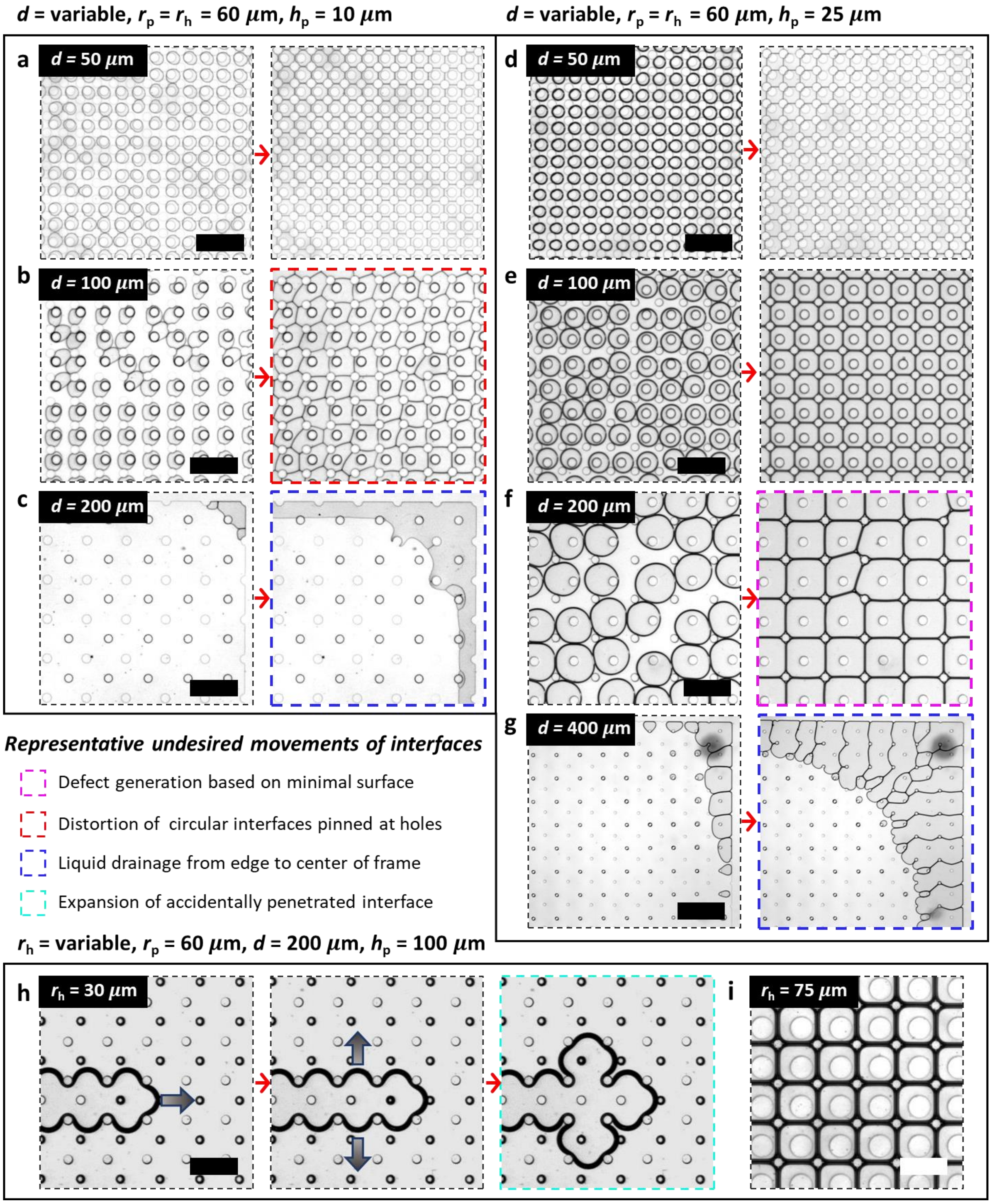

Supplementary Fig. 6. Aspects of moving liquid–air interfaces affected by geometric parameters during evaporation. (a–c) Images were acquired with varying d values when $r_p = r_h = 60$ μm and $h_p = 10$ μm. **(d–g)** Images were acquired with varying d values when $r_p = r_h = 60$ μm and $h_p = 25$ μm. **(h, i)** Images were acquired with different r_h values when $r_p = 60$ μm, $d = 200$ μm, and $h_p = 100$ μm. Dark arrows in (h) show direction of the interface expansion. The colored lines correspond to the representative undesired cases. The scale bars in (g) is 900 μm. Otherwise, the scale bars are 300 μm.

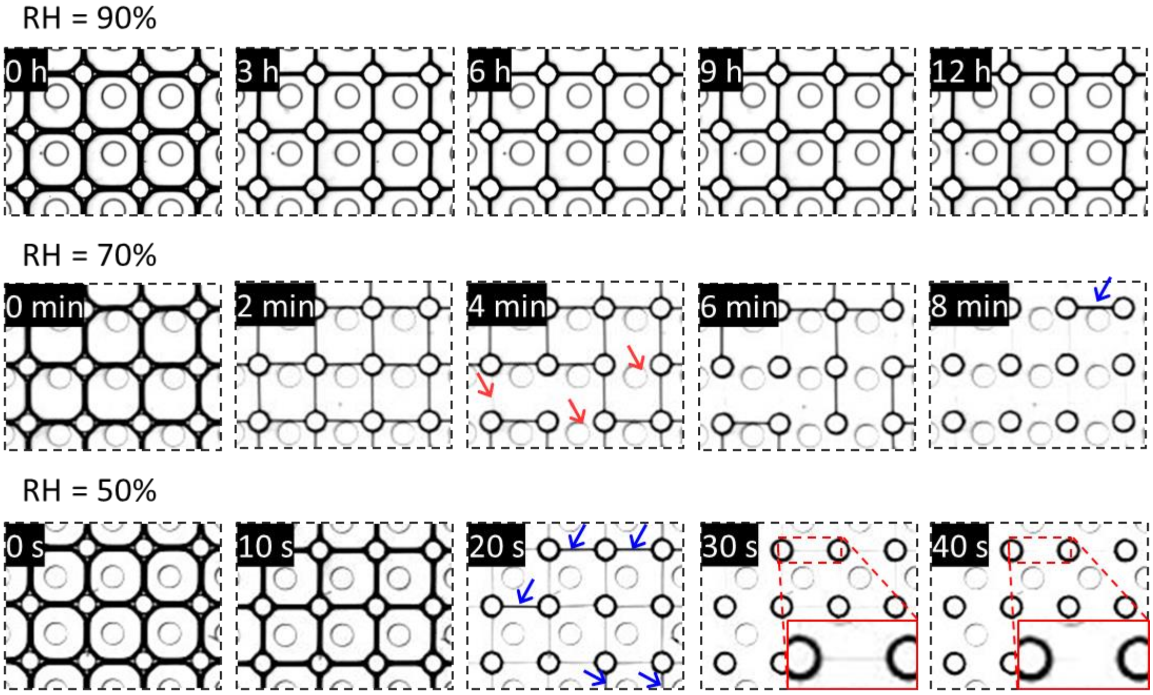

Supplementary Fig. 7. Time-sequential responses of the liquid patterns under different humidity conditions. Experiments were conducted at RH = 90, 70, and 50%, respectively. The blue and red arrows indicate those liquid films that have ruptured or not yet ruptured, respectively.

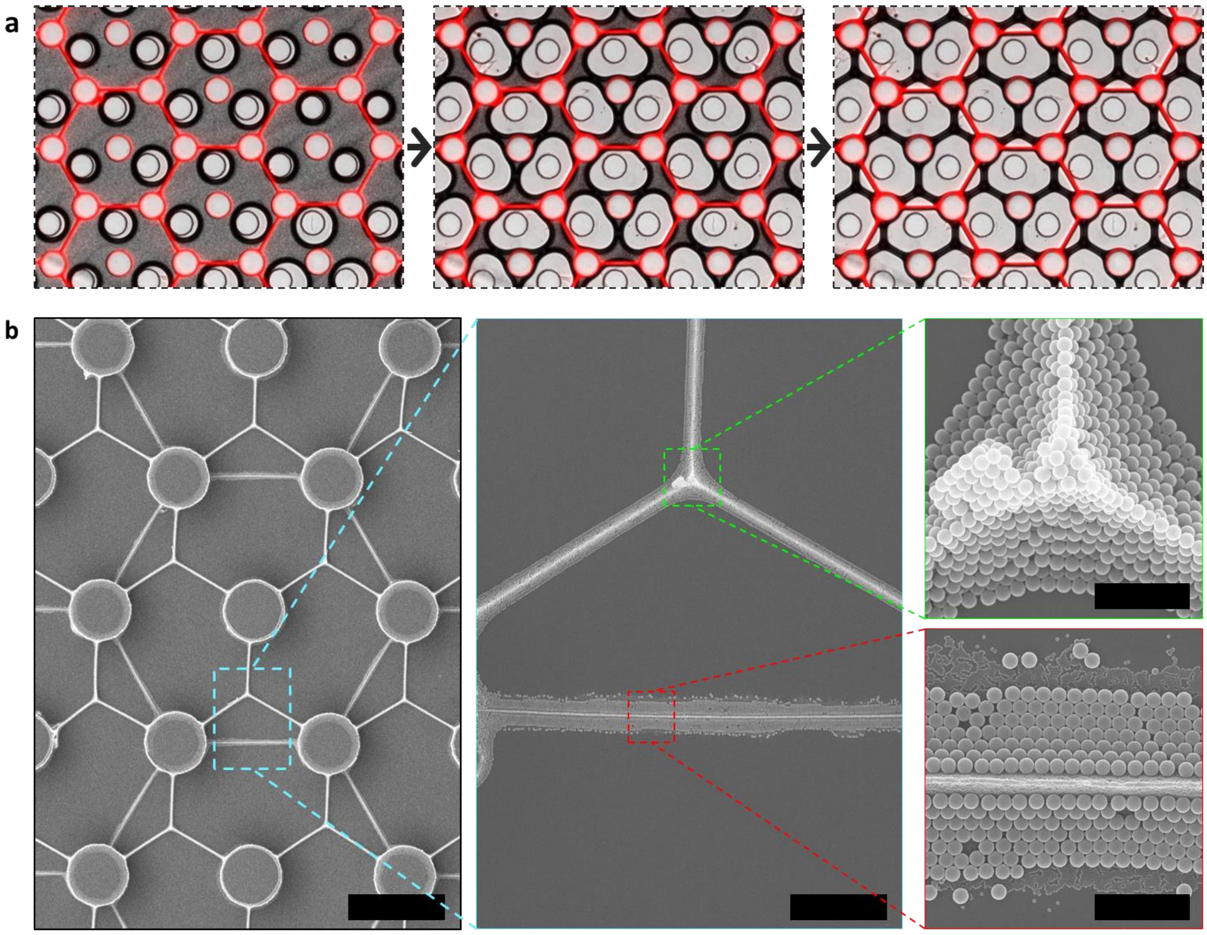

Supplementary Fig. 8. Integration of heterogenous nanomaterial patterns at several-micron scales. **(a)** Time-sequential images show the second MNLP process using a 15 mg mL^{-1} suspension of 500-nm GF PS NPs, after the first pattern was formed using a 10 mg mL^{-1} suspension of 200-nm RF PS NPs. This is the same as the experiment shown in Fig. 4c but differs in that high concentrations of liquid-mediated material samples were used to construct the micron-scale triangular structures. The RF images were merged with the time-lapse bright-field images. As shown in the middle image, the prepatterned micron-scale structures of the 200-nm RF PS NPs disturb the evaporative dynamics of the liquid-air interfaces. However, the well-ordered second patterns of the GF PS NPs were successfully produced, which can be attributed to the symmetrical mutual interaction between the interfaces. **(b)** SEM images show integrated patterns of heterogenous materials, produced by the process described in (a). Notably, the prepatterned micron-scale structures of the 200-nm RF PS NPs influenced the subsequent deposition of the other materials, resulting in a ridge of the 200-nm NPs covered with a monolayer of the 500-nm NPs. The scale bars are 100 μm (left), 20 μm (middle), and 3 μm (right).

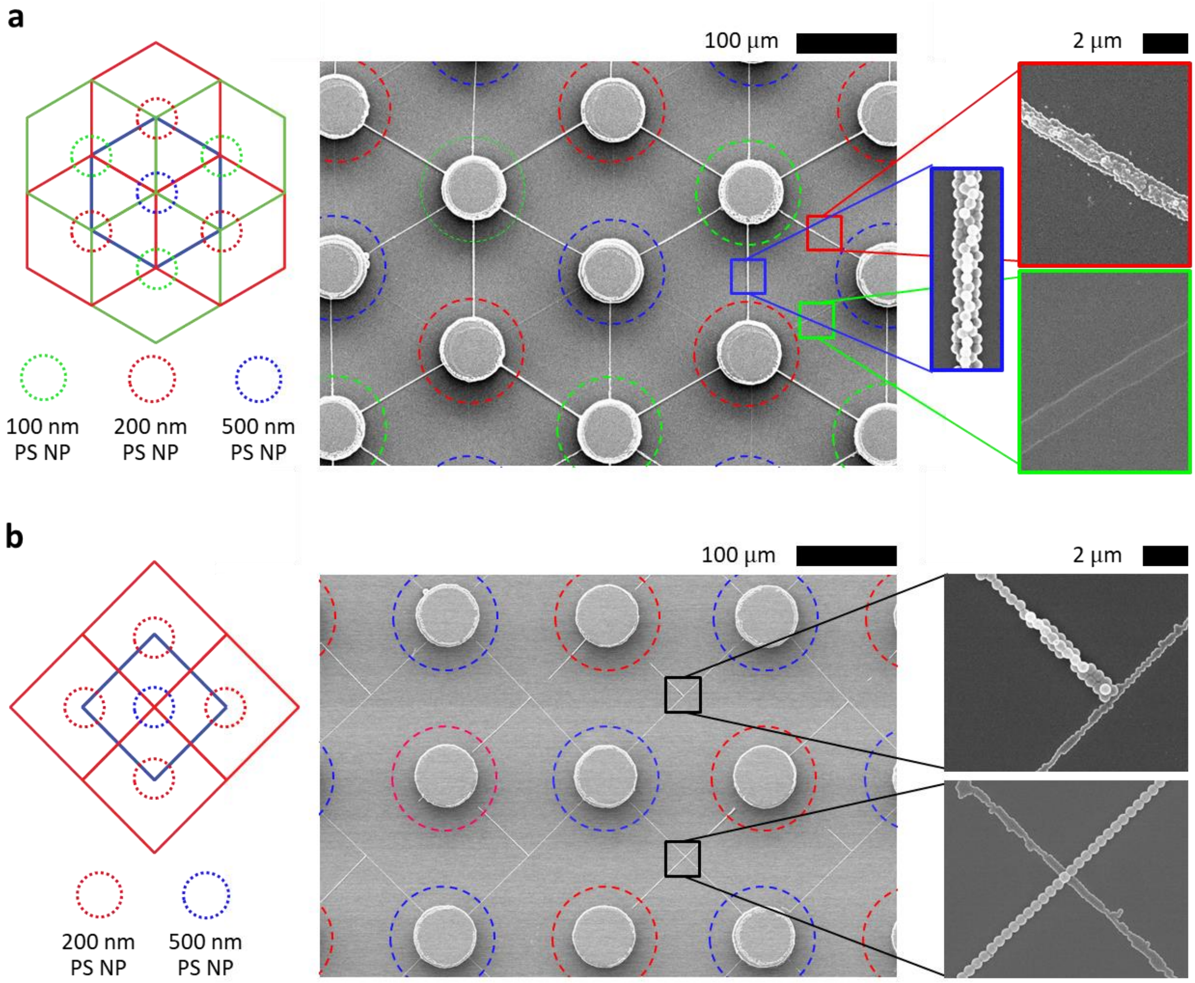

Supplementary Fig. 9. Multiple MNLP for complex integrated patterns of heterogeneous materials. (**a**) An integrated pattern of 100-nm GF, 200-nm RF, and 500-nm BF PS NPs. With multiple MNLP process, each fluorescent PS NPs was patterned into the same honeycomb-like pattern but on different positions. The integrated pattern forms remarkable periodic R-G-B triangular lines. Colored-dot circular lines indicate used configuration of the through-hole membrane. (**b**) An integrated pattern of 200-nm RF and 500-nm BF PS NPs. With multiple MNLP process, each fluorescent PS NP was patterned into the same grid pattern but on different positions. The integrated pattern remarkably forms perpendicularly crossed junctions of the heterogeneous materials. Colored-dot circular lines indicate the used configuration of the through-hole membrane.

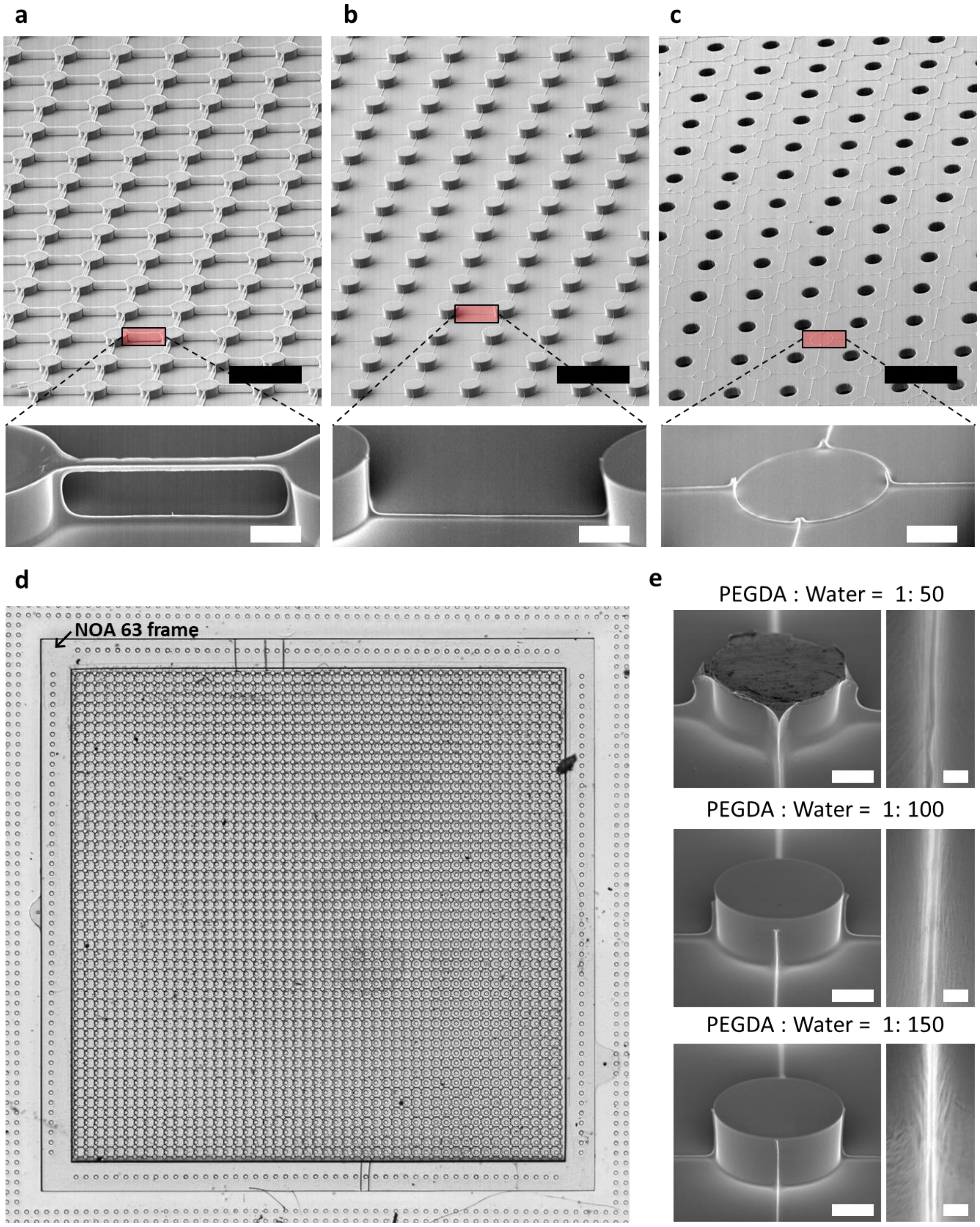

Supplementary Fig. 10. UV-cured pattern of PEGDA solution in a grid format. (**a**) SEM images of the 3D, large-area PEGDA structures for Case-I shown in Fig. 5e. The scale bars are 200 μm (top) and 20 μm (bottom). (**b, c**) SEM images of the 2D large-area PEGDA structures on the reservoir (left) and membrane (right) sides for Case-II shown in Fig. 5e. The scale bars are 200 μm (top) and 20 μm (bottom). (**d**) Curing of the entire liquid films in the 8 mm × 8 mm reservoir. (**e**) SEM images of the UV-cured PEGDA structures with respect to the mixing ratios of PEGDA to DI water. The scale bars are 20 μm (left) and 500 nm (right).

Supplementary Table

Experimental conditions	Post height (h_p)	Post radius (r_p)	Hole radius (r_h)	Distance btw. post (d)	Relative humidity	Post/hole array configuration	Result of the MNLP process
**Effect of r_p and d on minimal surface:** asymmetrical deformation of liquid-air interfaces by mutual contact between air-cells (Fig. 2b, c; Supplementary Fig. 4; Supplementary Fig. 5)	25 μm	30 μm	30 μm	50 μm	70%	4P1H	No defect
				200 μm			Small number of defects
		5, 15, 25, 35, 45, 55 μm		130 μm			Defect = 94.0, 29.6, 2.5, 1.8, 0.0, 0.0%, respectively
		20, 30, 40, 50, 60, 70 μm		200 μm			Defect= 39.0, 13.8, 3.9, 1.5, 0.5, 0.4%, respectively
		30 μm		100 μm		3P1H	High number of defects
		115 μm		100 μm			No defect
**Effect of d on structural stability of device:** collapse of the membrane (Fig. 2d; Supplementary Fig. 6d-g)	25 μm	30 μm	30 μm	50, 100, 200 μm	70%	4P1H	Uniform drainage
				400 μm			Drainage starts from holes at edge of frame
**1) Effect of h_p on liquid pinning at the hole:** distorted expansion of liquid-air interfaces (Supplementary Fig. 6a, b)	10 μm	30 μm	30 μm	50 μm	70%	4P1H	Slightly distorted interface → No defect
				100 μm			Highly distorted interface → Numerous defect
**2) Effect of h_p on surface tension:** membrane collapse (Supplementary Fig. 6c)				200 μm			Drainage starts from holes at edge of frame
**3) Effect of h_p depending on the r_h value** (Supplementary Fig. 6h, i)	100 μm	30 μm	30 μm	200 μm			No defect
			75 μm				No generation of foam
**Humidity control** (Fig. 2g Supplementary Fig. 7)	25 μm	30 μm	30 μm	100 μm	50%	4P1H	All rupture within 50 s
					70%		All rupture within 17 min
					90%		No rupture
**Configuration of a micropost array and a microhole array:** various liquid patterns (Fig. 3)	25 μm	30 μm	30 μm	100 μm	70%	Periodic removal of certain posts	Fig. 3d, e
						Periodic removal of certain holes	Fig. 3f, i
			60 and 75 μm			Double spaced hole array	Fig. 3g, h
			30 μm			Holes into a word	Fig. 3k
		N/A	75 μm	N/A		Without any post	Fig. 3j

Supplementary Table 1. Summary of geometrical and environmental parameters used for characterizing the MNLP.
