# Supplementary material for: Controlled open-cell two-dimensional liquid foam generation for micro- and nanoscale patterning of materials
Source: Nat Commun. 2019 Jul 19;10:3209. doi: 10.1038/s41467-019-11281-y (PMC6642206; doi:10.1038/s41467-019-11281-y)
Supplement: Supplementary file 3 — Description of Additional Supplementary Files [file 41467_2019_11281_MOESM3_ESM.pdf]

Description of Additional Supplementary Files

Supplementary Movie 1. Comparison between the engineered 2D liquid foam and the natural 2D liquid foam. Time-lapse microscopy of the MNLP process shows long-term responses of the liquid pattern at RH = 95, 90, and 50%, after generation of the liquid pattern at RH = 60%.

Supplementary Movie 2. Time-lapse microscopy of experiments shown in Fig. 3a–k. Radius (r_p) and height (h_p) of the posts are 30 µm and 25 µm, and distance between two neighboring posts (d) is 100 µm. Each of the time-lapse results is imaged at arbitrary time intervals.

Supplementary Movie 3. Time-lapse microscopy of experiments shown in Supplementary Fig. 8a. The radius (r_p) and height (h_p) of the posts are 30 µm and 25 µm, respectively, and the distance between the two neighboring posts (d) is 100 µm. Time-lapse images were obtained at an interval of 2 s. The running speed of the movie is 10 frames s^{-1}.
